# Supplementary material for: Stimulus encoding by specific inactivation of cortical neurons
Source: Nat Commun. 2024 Apr 12;15:3192. doi: 10.1038/s41467-024-47515-x (PMC11015011; doi:10.1038/s41467-024-47515-x)
Supplement: Supplementary file 1 — Supplementary Information [file 41467_2024_47515_MOESM1_ESM.pdf]

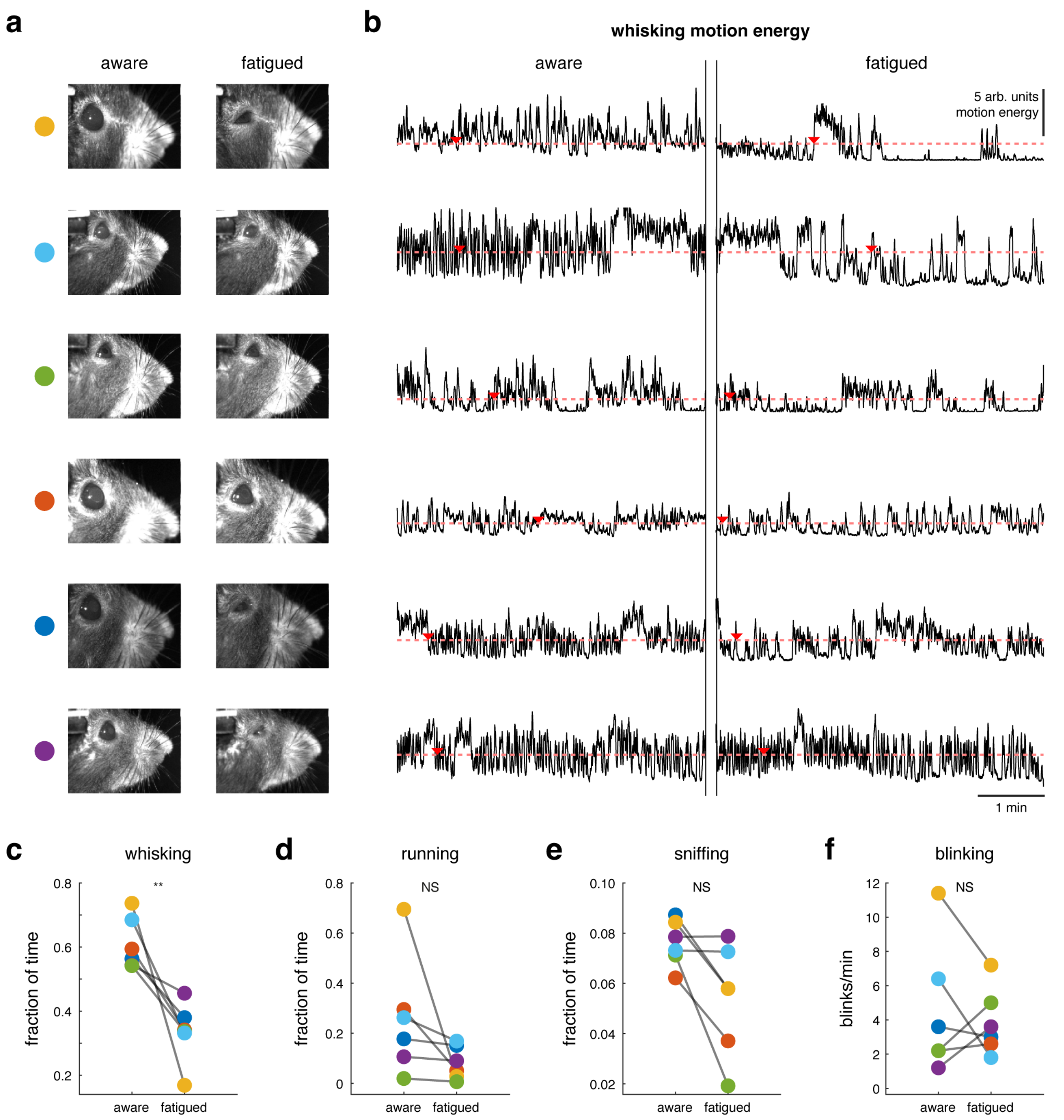

Supplementary Figure 1. Whisking reflects awareness.

a. Images of mice during their initial recording session (aware) and one hour later under the microscope (fatigued).

b. Whisker motion energy of mice in (a) during the initial session (aware) and a session one hour later (fatigued). The dashed line represents the average motion energy. The red triangles indicate the times of the pictures in (a), representing instances when the whisking motion energy was around the average, both in the aware and fatigued condition.

c. Fraction of the session time in which mice were whisking (above the average motion energy), in both aware and fatigued conditions. Mouse whisking can determine awareness behavior.

d. Fraction of the session time in which mice were running (running speed > 1 cm/s), in both aware and fatigued conditions. Although running speed decreases during mouse fatigued periods, sometimes mice do not run during awareness periods. Thus, running speed is not enough to characterize awareness.

e. Fraction of the session time in which mice were sniffing (complete displacement of the nose), in both aware and fatigued conditions.

f. Number of blinks per minute for each mouse, in both aware and fatigued conditions. From (c) to (f), each data point represents a single mouse, across $n = 6$ mice. Although data are paired, we used two-sided Mann-Whitney test to identify which behavior best characterizes periods of awareness (NS = Not Significant; $^{**}p = 0.002$). Source data are provided as a Source Data file.

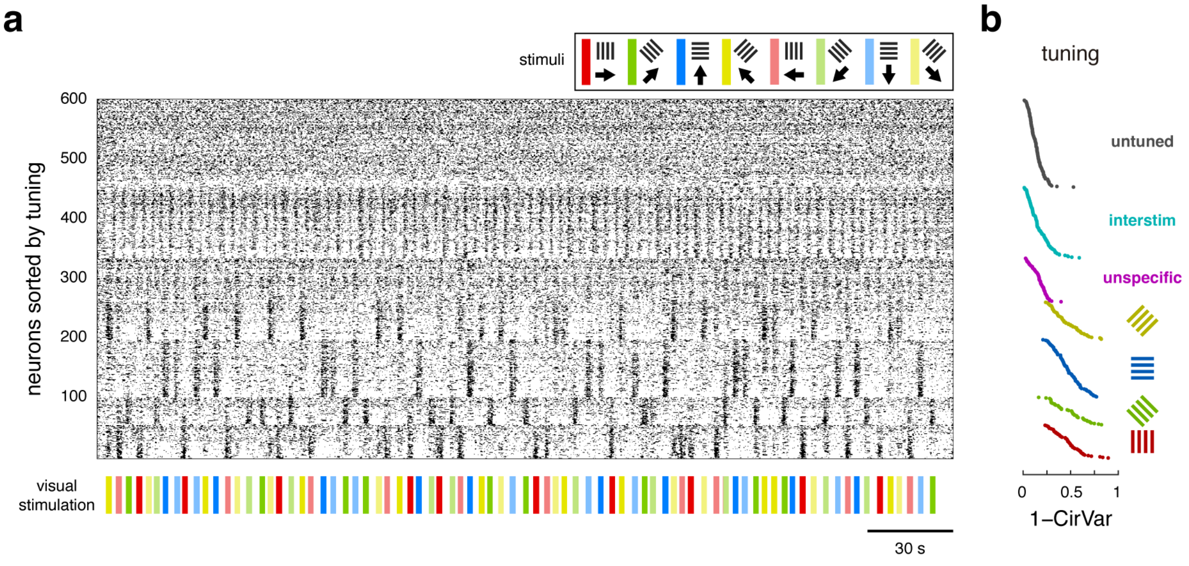

Supplementary Figure 2. Tuning of individual neurons.

a. Neuronal activity during a five-minute session of visual stimulation (as in Figure 1d) with neurons sorted by tuning to a single orientation (4 orientations), unspecific tuning, interstimulus activity (interstim), and untuned responses.

b. Orientation selectivity indices (based on circular variance) of each neuron in (a) and their respective subdivided categories.

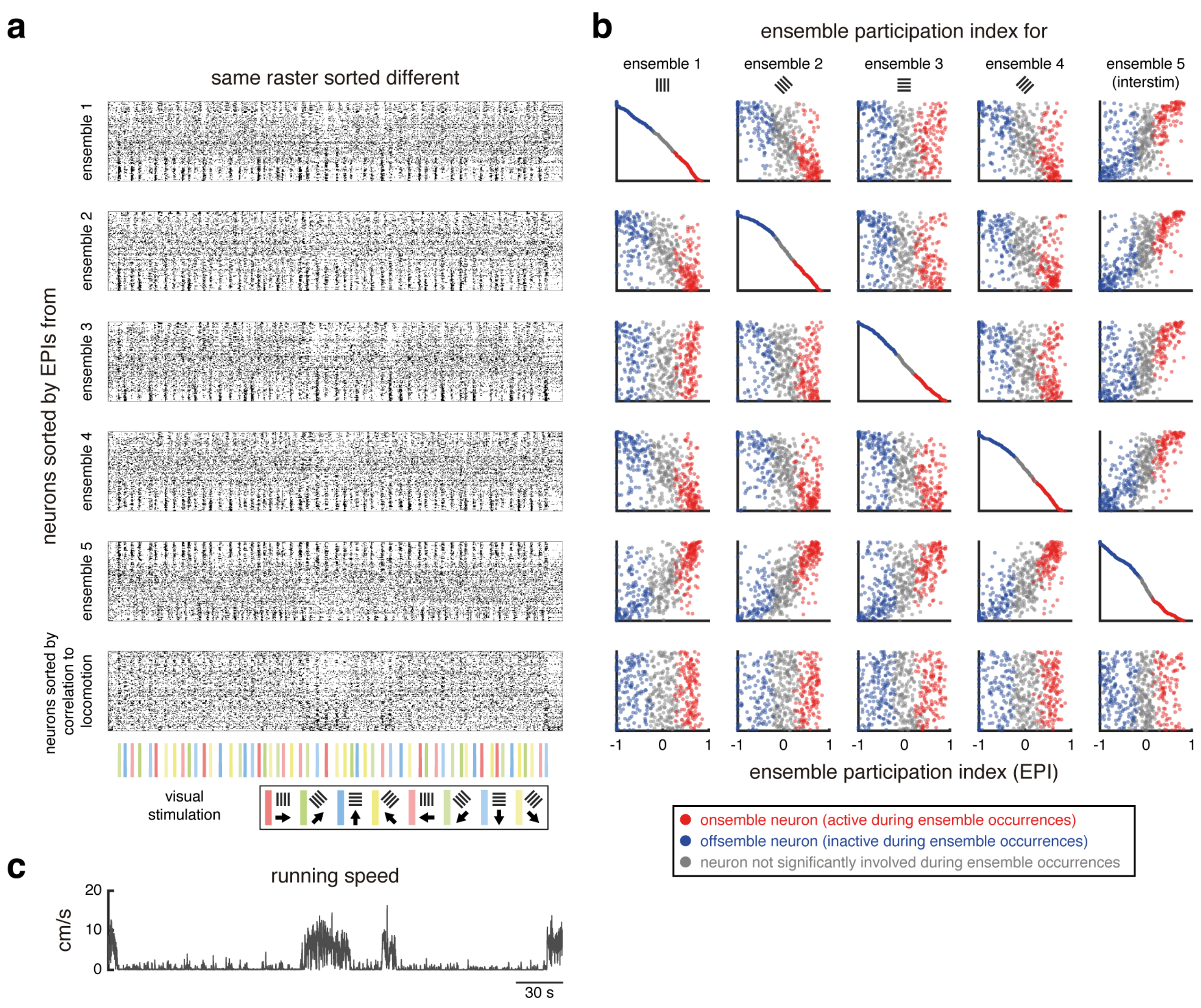

Supplementary Figure 3. Participation indices for different ensembles.

a. The same population activity raster plot with neurons sorted based on their EPIs for each ensemble and also sorted by their correlation to the mouse running speed. Visual stimulus orientations and their timestamps are shown below.

b. EPIs of all neurons computed for each ensemble, sorted as shown in (a). In this example, every neuron has five EPIs, one for each ensemble. Neurons can be significantly activated (red), significantly inactivated (blue), or not involved (gray) during ensemble occurrences. Typically, neurons exhibit varying participation values for each ensemble.

c. Mouse running speed during the five-minute session of visual stimulation.

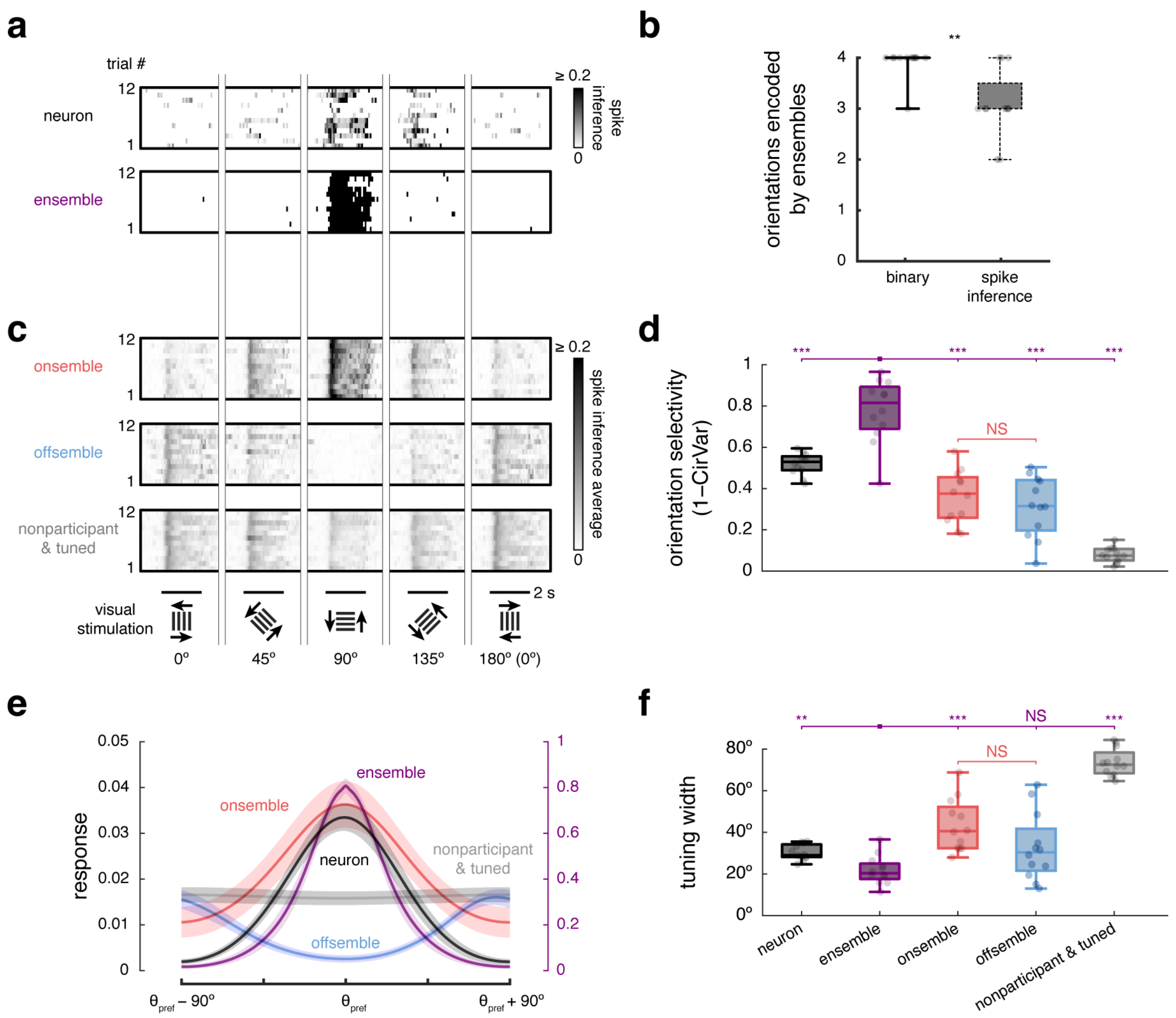

Supplementary Figure 4. Orientation selectivity using spike inference instead of binary thresholded neuronal activity.

a. Similar example as in Figure 5 but analyzing the responses using the neuronal spike inference instead of binary spikes. Responses to trials of four different orientations for a neuron (spike inference) and an ensemble (identified using neuronal spike inference), both tuned to the same preferred orientation (in this case, 90º). For enhancing presentation, trials at 0º orientation are duplicated in 180º orientation (mirrored). Spike inference is presented in arbitrary units.

b. Comparison of the number of orientations encoded by ensembles between using binary raster spikes and nonbinary spike inference raster to identify ensembles. Two-sided Wilcoxon test (n = 12 mice; $^{**}p = 0.004$).

c. Example responses, across all trials, of onsemble, offsemble, and the nonparticipant-tuned population, as in Figure 5d. Onsemble, offsemble, and nonparticipant-tuned population responses are presented as spike inference average.

d. Orientation selectivity (extracted from the circular variance) for individual neurons, ensembles, onsembles, offsembles, and the nonparticipant-tuned population. Pairwise two-sided Wilcoxon test between groups was conducted. Ensembles were more selective than single neurons ($p = 9 \times 10^{-4}$) and more selective than the other groups ($p = 5 \times 10^{-4}$). Each data point represents the average within each mouse across n = 12 mice.

e. Orientation tuning curves fitted for individual neurons, ensembles, onsembles, offsembles, and the nonparticipant-tuned population. Responses of neurons, onsembles, offsembles, and nonparticipant populations are spike inference average (left scale), and ensemble responses are average activation (right scale). Lines and shaded regions represent the mean ± SEM across n = 12 mice. Tuning curves are plotted with the preferred orientation (θ_{pref}) at the center.

f. Tuning widths, extracted from orientation tuning curves, for individual neurons, ensembles, onsembles, offsembles, and the nonparticipant-tuned population. Pairwise two-sided Wilcoxon test between groups was conducted. Ensembles exhibited a narrower tuning width compared to single neurons ($p = 0.002$) and the other groups ($p = 5 \times 10^{-4}$), except for offsembles ($p = 0.05$). Each data point represents the average within each mouse across n = 12 mice. NS = Not Significant, $^{**}p < 0.01$, and $^{***}p < 0.001$. The center of boxplots represents the median, the bounds of the boxes correspond

to the first and third quartiles, and the whiskers extend to the minimum and maximum datapoint values. Source data are provided as a Source Data file.

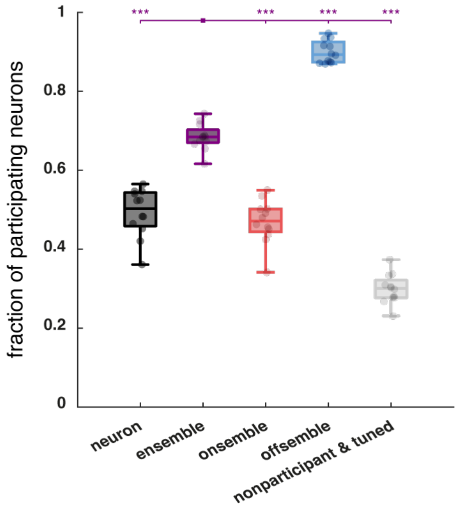

Supplementary Figure 5. Neuronal participation in response to preferred orientation.

Comparison between neuronal participation across different groups. The fraction of activated tuned neurons (neuron), the fraction of activated onsemble neurons (onsemble), the fraction of inactivated neurons of the offsemble, the fraction ensemble neurons participating (including activated onsemble neurons and inactivated offsemble neurons), and the fraction of nonparticipant-tuned neurons. Each datapoint represents the average within each mouse across n = 12 mice. Pairwise two-sided Wilcoxon test between groups was conducted (***$p = 5 \times 10^{-4}$). The center of boxplots represents the median, the bounds of the boxes correspond to the first and third quartiles, and the whiskers extend to the minimum and maximum datapoint values. Source data are provided as a Source Data file.

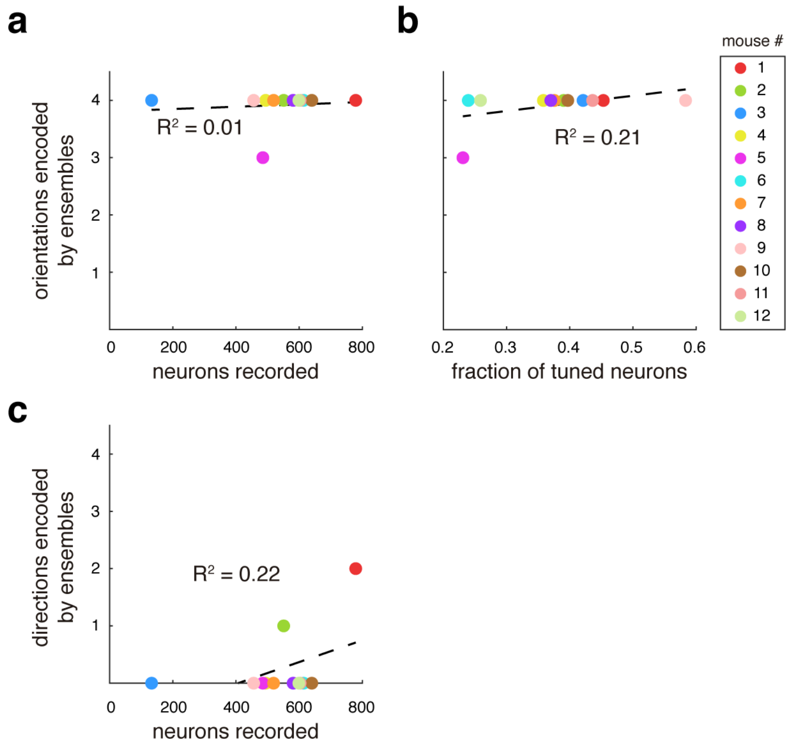

Supplementary Figure 6. Correlation between ensemble encoding and neurons recorded or tuned.

a. Exploring the correlation between the number of recorded neurons and the number of ensembles tuned to orientated drifting gratings (maximum number of orientations: 4). The dashed line represents their linear relationship.

b. Exploring the correlation between the fraction of neurons tuned to orientations and the orientations encoded by ensembles. The dashed line represents their linear relationship.

c. Exploring the correlation between the number of recorded neurons and the number of ensembles tuned to the direction of drifting gratings. The dashed line represents their linear relationship. Each datapoint represents a mouse across n = 12 mice. Source data are provided as a Source Data file.

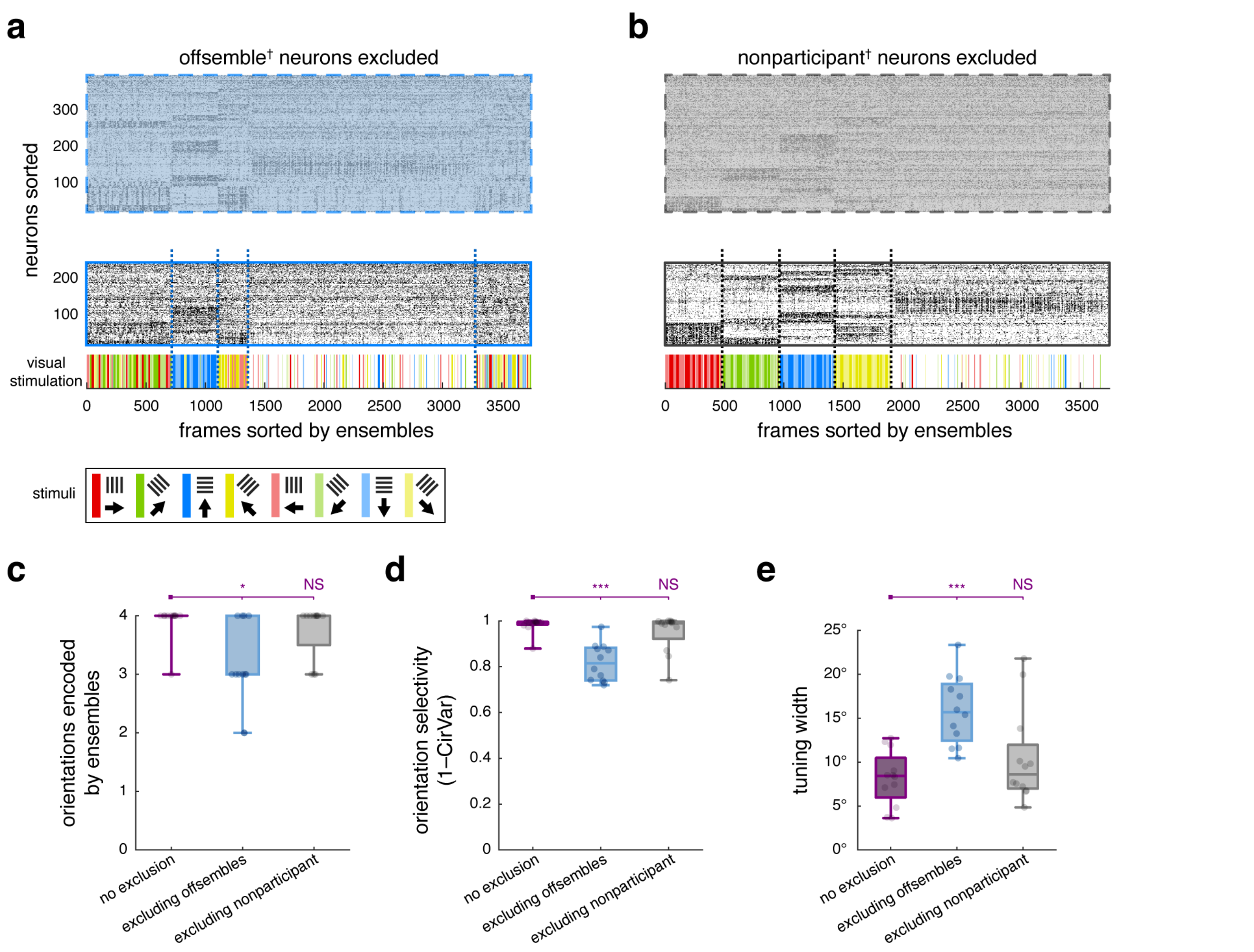

Supplementary Figure 7. Removal of offsemble neurons reduces ensemble orientation selectivity.

a. Same example as in Figure 1 with the neuronal population of offsemble neurons excluded from the analysis (top) and the remaining population activity (bottom) analyzed to find neuronal activity patterns (ensembles). The frames of the activity are sorted by ensembles. Visual stimulation is indicated at the bottom. Offsemble† neurons can be at the same time onsemble neurons or nonparticipant neurons in a different ensemble.

b. Same as in (a) but with the neuronal population of nonparticipant neurons excluded from the analysis (top) and the remaining population activity (bottom) analyzed to find ensembles. Frames are sorted by

ensembles. Visual stimulation is indicated at the bottom. Nonparticipant† neurons can be at the same time onsemble or offsemble neurons in a different ensemble.

c. Number of ensembles tuned to orientated drifting gratings using the entire population recorded remained similar when excluding nonparticipant neurons (p = 0.5), but decreased when excluding offsembles (p = 0.02). Each datapoint represents a mouse across n = 12 mice Pairwise two-sided Wilcoxon test was performed between groups.

d. Orientation selectivity of ensembles found using the entire population recorded (as in Figure 5e) remained similar when excluding nonparticipant neurons (p = 0.1), but decreased when excluding offsembles ($p = 5 \times 10^{-4}$). Each datapoint represents the average within each mouse across n = 12 mice. Pairwise two-sided Wilcoxon test was performed between groups.

e. Tuning width of ensembles found using the entire population recorded (as in Figure 5g) remained similar when excluding nonparticipant neurons (p = 0.3), but increased when excluding offsembles ($p = 5 \times 10^{-4}$). Each datapoint represents the average within each mouse across n = 12 mice. Pairwise two-sided Wilcoxon test was performed between groups. The center of boxplots represents the median, the bounds of the boxes correspond to the first and third quartiles, and the whiskers extend to the minimum and maximum datapoint values. NS = Not Significant, *$p < 0.05$, and ***$p < 0.001$. Source data are provided as a Source Data file.

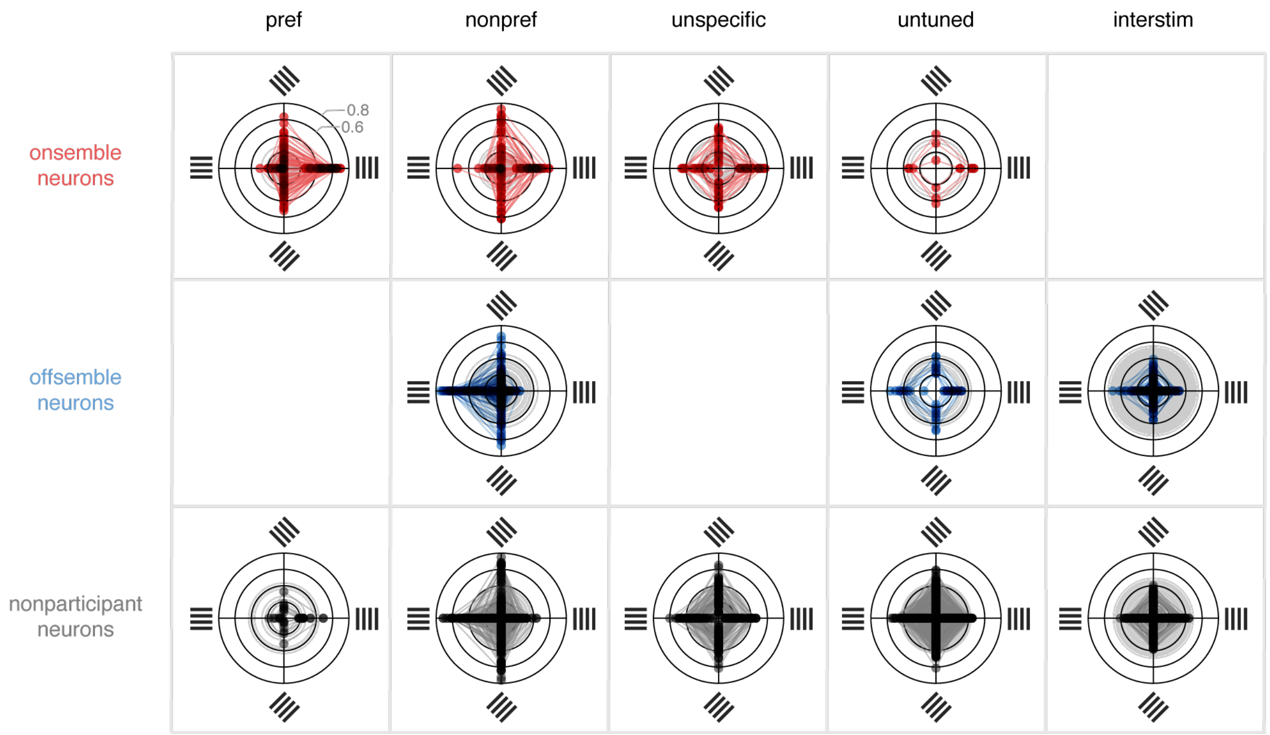

Supplementary Figure 8. Polar plots of responses of individual neuron to stimulus orientations. Single neuron responses to stimulus orientations (as shown in Figure 7a). Responses are grouped into onsemble, offsemble, and nonparticipant neurons. Each group is further subdivided based on single-neuron classification tuning: preferred orientation (pref), nonpreferred orientation (nonpref), unspecific, untuned, and interstimulus tuned (interstim). Dots represent the amplitude of the average responses for each neuron, lines connect the responses of the same neuron, and gray background circles indicate the average activity during interstimulus periods.
