# Supplementary material for: Stimulus encoding by specific inactivation of cortical neurons
Source: Nat Commun. 2024 Apr 12;15:3192. doi: 10.1038/s41467-024-47515-x (PMC11015011; doi:10.1038/s41467-024-47515-x)
Supplement: Supplementary file 3 — Reporting Summary [file 41467_2024_47515_MOESM3_ESM.pdf]

nature portfolio

Corresponding author(s): Jesús Pérez-Ortega

Last updated by author(s): Mar 6, 2024

Reporting Summary

Nature Portfolio wishes to improve the reproducibility of the work that we publish. This form provides structure for consistency and transparency in reporting. For further information on Nature Portfolio policies, see our Editorial Policies and the Editorial Policy Checklist.

Statistics

For all statistical analyses, confirm that the following items are present in the figure legend, table legend, main text, or Methods section.

n/a	Confirmed	
☐	☒	The exact sample size (n) for each experimental group/condition, given as a discrete number and unit of measurement
☐	☒	A statement on whether measurements were taken from distinct samples or whether the same sample was measured repeatedly
☐	☒	The statistical test(s) used AND whether they are one- or two-sided<br>*Only common tests should be described solely by name; describe more complex techniques in the Methods section.*
☒	☐	A description of all covariates tested
☒	☐	A description of any assumptions or corrections, such as tests of normality and adjustment for multiple comparisons
☐	☒	A full description of the statistical parameters including central tendency (e.g. means) or other basic estimates (e.g. regression coefficient) AND variation (e.g. standard deviation) or associated estimates of uncertainty (e.g. confidence intervals)
☐	☒	For null hypothesis testing, the test statistic (e.g. F, t, r) with confidence intervals, effect sizes, degrees of freedom and P value noted<br>*Give P values as exact values whenever suitable.*
☒	☐	For Bayesian analysis, information on the choice of priors and Markov chain Monte Carlo settings
☐	☒	For hierarchical and complex designs, identification of the appropriate level for tests and full reporting of outcomes
☒	☐	Estimates of effect sizes (e.g. Cohen's d, Pearson's r), indicating how they were calculated

Our web collection on statistics for biologists contains articles on many of the points above.

Software and code

Policy information about availability of computer code

Data collection	- Prairie View V5.5 (from Bruker) was used to control the two-photon microscope and record calcium imaging data simultanously with voltage recordings.<br>- MouSee V1.0 (Custom-made code in Matlab, https://doi.org/10.5281/zenodo.7765050) was used to generate and present visual stimuli on a monitor in front of a mouse. This program generates a voltage output through a data board (USB-6008, National Instruments) for every stimulus presented in the monitor.<br>- IC Capture software V2.5 (from The Imaging Source) was used to acquire and store images of the mouse face during the experiment.
Data analysis	- Xsembles2P V1.1 (Custom-made code in Matlab, https://doi.org/10.5281/zenodo.8423311) was used to:<br>1. Read raw videos.<br>2. Read external voltage recording files.<br>3. Perform registration (based on animal locomotion).<br>4. Find active neurons.<br>5. Get calcium signals.<br>6. Do spike inference.<br>7. Get population activity (binary raster).<br>8. Find ensembles (onsembles and offsembles).<br>- MoussionEnergy V1.0 (Custom-made code in Matlab, https://doi.org/10.5281/zenodo.8422691) was used to analyze mouse facial behaviours such as whisking, blinking, and sniffing.<br>- Custome-made code in Matlab to compute different known measures (orientation selectivity, tuning, etc.)

For manuscripts utilizing custom algorithms or software that are central to the research but not yet described in published literature, software must be made available to editors and reviewers. We strongly encourage code deposition in a community repository (e.g. GitHub). See the Nature Portfolio guidelines for submitting code & software for further information.

Data

Policy information about availability of data

All manuscripts must include a data availability statement. This statement should provide the following information, where applicable:

- Accession codes, unique identifiers, or web links for publicly available datasets
- A description of any restrictions on data availability
- For clinical datasets or third party data, please ensure that the statement adheres to our policy

The experimental preprocessed dataset and results generated in this study have been deposited in the Dryad repository (https://doi.org/10.5061/dryad.w3r2280w7).

Research involving human participants, their data, or biological material

Policy information about studies with human participants or human data. See also policy information about sex, gender (identity/presentation), and sexual orientation and race, ethnicity and racism.

Reporting on sex and gender	Our study did not involve human participants.
Reporting on race, ethnicity, or other socially relevant groupings	Our study did not involve human participants.
Population characteristics	Our study did not involve human participants.
Recruitment	Our study did not involve human participants.
Ethics oversight	Our study did not involve human participants.

Note that full information on the approval of the study protocol must also be provided in the manuscript.

Field-specific reporting

Please select the one below that is the best fit for your research. If you are not sure, read the appropriate sections before making your selection.

☒ Life sciences ☐ Behavioural & social sciences ☐ Ecological, evolutionary & environmental sciences

For a reference copy of the document with all sections, see nature.com/documents/nr-reporting-summary-flat.pdf

Life sciences study design

All studies must disclose on these points even when the disclosure is negative.

Sample size	Our sample size of twelve mice was appropiate for our experimental design and allowed us to obtain enough ensembles to get statistically significant results in our analysis. We also took into consideration ethical reasons and minimized the number of mice used in our study.
Data exclusions	Eight mice were excluded from our study because they were partially recorded in primary visual cortex, as confirmed by wide-field GCaMP imaging after the completion of the experiment. When we analyze the data from these mice, less than 20% of the recorded neurons were not visually responsive. Thus, to ensure the accuracy and validity of our data, we decided to exclude these mice from our analysis.
Replication	We have seen 100% replicability in all awake mice recorded in primary visual cortex.
Randomization	Randomization does not apply in this study because we were not comparing between different groups of mice.
Blinding	Blinding was not relevant in this study because we were not comparing between different groups of mice.

Reporting for specific materials, systems and methods

We require information from authors about some types of materials, experimental systems and methods used in many studies. Here, indicate whether each material, system or method listed is relevant to your study. If you are not sure if a list item applies to your research, read the appropriate section before selecting a response.

Materials & experimental systems

n/a	Involved in the study
☒	☐ Antibodies
☒	☐ Eukaryotic cell lines
☒	☐ Palaeontology and archaeology
☐	☒ Animals and other organisms
☒	☐ Clinical data
☒	☐ Dual use research of concern
☒	☐ Plants

Methods

n/a	Involved in the study
☒	☐ ChIP-seq
☒	☐ Flow cytometry
☒	☐ MRI-based neuroimaging

Animals and other research organisms

Policy information about studies involving animals; ARRIVE guidelines recommended for reporting animal research, and Sex and Gender in Research

Laboratory animals	We performed experiments in adult transgenic mice (Slc17a7-IRES2-Cre, JAX stock # 023527) crossed with TIGRE2.0 Ai162 (TIT2L-GC6s-ICL-tTA2, JAX stock # 031562) maintained in C57BL/6 J congenic background.<br>Ages of the 12 mice at the experiment day were: 8, 10, 11, 11, 12, 12, 16, 20, 20, 26, 69, and 69 weeks old.
Wild animals	This study did not involve wild type mice.
Reporting on sex	We did not analyze the data based on animal sex, as we aimed to investigate the overall neuronal responses during visual stimuli. We performed experiments in nine female mice and three male mice. We believe that sex differences would not significantly impact the results of our study.
Field-collected samples	This study did not involve field-collected samples.
Ethics oversight	All experimental procedures were conducted in accordance with the US National Institutes of Health and Columbia University Institutional Animal Care and Use Committee.

Note that full information on the approval of the study protocol must also be provided in the manuscript.
